# Uterine mullerian adenosarcoma with sarcomatous overgrowth fatal recurrence within two weeks of diagnosis: a case report

**DOI:** 10.1186/1752-1947-1-103

**Published:** 2007-09-25

**Authors:** Mirna H Farhat, Elie M Hobeika, Ghada Moumneh, Anwar H Nassar

**Affiliations:** 1 Department of Internal Medicine, American University of Beirut Medical Center, Beirut, Lebanon; 2 Department of Obstetrics and Gynecology, American University of Beirut Medical Center, Beirut, Lebanon; 3 Department of Pathology, Rafic Hariri University Hospital, Beirut, Lebanon

## Abstract

Mullerian adenosarcoma with sarcomatous overgrowth (MASO) is a rare variant of uterine sarcomas, associated with postoperative recurrence, metastases and a fatal outcome. The mean age at diagnosis is 54.5 years. A 37-year-old nullipara presented with irregular vaginal bleeding, a normal pelvic examination, and an initially negative ultrasound. Repeat ultrasound one month later revealed an 11-cm heterogeneous pelvic mass. She underwent total abdominal hysterectomy and bilateral salpingo-oophorectomy. Pathology confirmed uterine MASO. Computed tomography 2 weeks postoperatively showed a huge mass compatible with recurrence. Patient died 2 weeks later. MASO is rarely diagnosed in women in their 4th decade. This case stresses that these aggressive tumors should be considered in the differential of patients with vaginal bleeding and pelvic masses irrespective of their age.

## Background

Uterine sarcomas generally account for less than 4% of all uterine malignancies with the subtype adenosarcoma comprising only 8% of cases, reflecting the rarity of this entity [1]. Mullerian adenosarcoma with sarcomatous overgrowth (MASO) is a rare variant of uterine adenosarcomas first described in 1989 [2] and usually occurs in the sixth decade of life [3]. We report a 37-year-old woman who was diagnosed with MASO and died within a month of diagnosis.

## Case history

A 37-year-old nullipara, previously healthy, presented with a 3-month history of irregular heavy menstrual bleeding. Ultrasound of the pelvis showed normal pelvic structures and the absence of any masses. Shortly afterwards, she started to report dull left lower quadrant abdominal pain, increased abdominal girth, and malodorous greenish vaginal discharge. A repeat ultrasound performed a month later showed a huge amount of abdominal ascites and a heterogeneous formation measuring ~11 cm in the pelvis. Liver, pancreas, kidney, spleen and bladder were normal. Abdominal tap was done and bloody fluid was aspirated. Cytology of the fluid showed numerous malignant cells. A computed tomography (CT) of the abdomen and pelvis revealed a big heterogeneous tumor in the pelvis with thickening and irregularity of the peritoneum.

The patient underwent a total abdominal hysterectomy with bilateral salpingooophorectomy and omentectomy. A 3-kg tumor floating in the peritoneum was evacuated. The tumor revealed abundant surface ulceration associated with necrosis.

**Figure 1:**
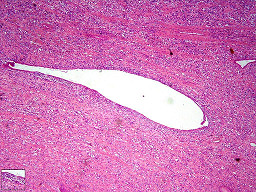
Low power: A biphasic tumor composed of benign epithelial elements and a sarcomatous stroma.

**Figure 2:**
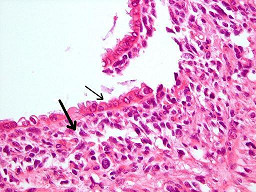
High Power: Many of these large benign cystically dilated glands are cuffed by packed hypercellular layer of malignant stromal cells (thick arrow). The epithelial component consists of scattered irregular dilated benign endometrial glands lined by a single layer of low columnar epithelium (thin arrow).

**Figure 3:**
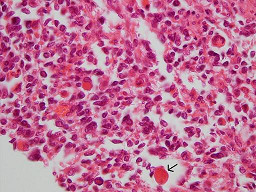
Note the scattered plumped large round cells with deeply eosinophilic cytoplasm and eccentric nuclei that resemble rhabdomyoblasts (arrow).

Histology of the polypoid mass revealed a biphasic tumor composed of benign epithelial elements and a sarcomatous stroma (Figure 1). Most of these large benign cystically dilated endometrial glands were cuffed by a packed hypercellular layer of malignant stromal cells (Figure 2). The stromal component arised from the endometrial stroma and was admixed with foci of undifferentiated cells with large polygonal, elongated and round hyperchromatic nuclei with very high mitotic figures (up to 18/10 HPF), many of which were bizarre and atypical. Also noted were abundant scattered plumped large round cells with deeply eosinophilic cytoplasm and eccentric nuclei resembling rhabdomyoblasts (Figure 3). Areas of pure sarcoma were abundant. The stroma contained abundant network of capillary-type blood vessels. Foci of necrosis and abundant hemorrhage were seen. Abundant lymphatic permeation of small sarcomatous tissue was noted within the tumor bulk. The tumor invaded into the inner half of the myometrium and focal extension into the stroma of the endocervix was noted.

A panel of immunohistochemical stains, including smooth muscle actin (SMA), muscle specific actin (MSA), desmin, S-100, CD10, multiCK, epithelial membrane antigen (EMA), and vimentin were performed and cells were positive for MSA and vimentin. The findings were consistent with MASO. The sarcoma was of the undifferentiated endometrial stromal type with rhabdomyosarcomatous differentiation. Two weeks later, the patient's abdomen increased in size again. Ultrasound of the abdomen showed a huge mass, and tumor recurrence was considered. Spiral CT revealed extensive peritoneal carcinomatosis. Patient died after 2 weeks of disease recurrence before adjuvant therapy could be administered.

## Discussion

Mullerian adenosarcomas of the uterus rarely present in premenopausal women. It is even more unusual to diagnose the rare variant MASO in this age group with a reported mean age at diagnosis of 54.5 years [3]. The most common presenting symptom is abnormal vaginal bleeding (71%) ranging from spotting to menorrhagia, as in our patient. One can argue that a uterine curettage for the 3-month history of heavy and irregular vaginal bleeding would have resulted in an earlier diagnosis of her disease. However, whether this would have had any implications on the prognosis remains unanswered.

These tumors can present as a pelvic mass (37%), uterine polyp (22%), or an enlarged uterus (22%) [4]. Pain, foul smelling vaginal discharge, or symptoms of pelvic pressure have also been reported. Risk factors that have been associated with an increased risk of uterine adenosarcoma include unopposed estrogen stimulation [5], long term oral contraceptive use [6], and prolonged use of tamoxifen for breast cancer [7,8]. Our patient had none of these risk factors.

A review by Krivak et al revealed 37 cases of MASO described in the literature up to 2001 [3]. MASO has a benign glandular component and a malignant sarcomatous component that constitutes more than 25% of the tumor [9]. It may have a heterologous sarcoma component; that is the sarcomatous component is derived from tissue not native to the uterus, such as cartilage, osteoid and striated muscle [10], of which the rhabdomyosarcoma is the most common as was noted in our patient. The presence of heterologous elements, especially rhabdomyosarcoma may represent a more clinically aggressive tumor [11]. The histologic finding of sarcomatous overgrowth is a predictor of poor prognosis while deep myometrial invasion is considered a predictor of aggressive behavior [12].

ASSO are considered high grade, have a high mitotic rate, and are DNA aneuploid with an S-phase fraction >10% [13]. The fact that the ultrasound done at onset of symptoms failed to detect any pelvic or abdominal mass, while an ultrasound done a month later showed a large 11 cm mass reflects the high mitotic rate observed in such tumors, and raises the question of how frequent should radiological and clinical follow up be performed in patients with high grade aggressive tumors such as MASO. In contrast to typical Mullerian adenosarcomas of the uterus, MASO are more aggressive tumors, frequently associated with postoperative recurrence (25%) or metastases, and have a fatal outcome [8]. Recurrence and metastasis were reported to occur even in early stages of the disease. In cases of recurrence, the interval between detection of the initial and the recurring tumors is no less than 9 months [9].

Cornerstone of therapy of uterine sarcomas remains surgery. Total abdominal hysterectomy with bilateral salpingo-oophorectomy (TAHBSO) can be curative if disease was confined to the uterus with five-year survival approaching 50% [14]. Adjuvant radiotherapy appears to have a role in better pelvic control and decrease in local recurrence of the tumor [15]. However, improvement in survival is still controversial [15,16]. Data regarding the benefit of adjuvant chemotherapy is very controversial, especially with the paucity of studies and lack of control. The chemotherapeutic agents consist of either doxorubicin or a combination of cisplatin and ifosfamide with mesna [3]. Intraperitoneal disease may be treated with whole abdomen radiation or chemotherapy. Ovarian conservation in women of reproductive age with Mullerian adenocarcinoma is an option in cases of non-metastatic disease, confined to the uterus [17]. The overall median survival time of patients with uterine MASO is 13 months [2]. Survival of patients with MASO can be as short as 1 month even after surgery and adjuvant therapy [3]. Our patient's tumor recurred two weeks after the resection of the initial mass which highlights the necessity of extensive debulking surgery with removal of all possible tumor implants and the need to initiate postoperative adjuvant therapy for better control of disease and prevention of recurrence. Whether the 2-week delay in initiation of adjuvant therapy, or the failure to remove all lesions by the debulking surgery resulted in early recurrence of the disease in our patient cannot be known.

The rarity of such tumors in young age groups makes the clinical suspicion very low, resulting in delay in making the diagnosis, and with highly proliferative tumors, rate of complications is expected to increase as seen in our patient who presented with extensive peritoneal metastasis. Whether a repeat ultrasound performed at a shorter interval would have detected the tumor at an earlier stage before it grows and spreads is to be considered. In addition, due to the rarity of adenosarcomas there is no consensus regarding therapy. A study dealing with the time of initiation of postoperative adjuvant therapy in relation to the interval to tumor recurrence is further needed to outline recommendations regarding promptness of adjuvant therapy and its effect on survival, tumor progression, and disease free period.

In conclusion, gynecologists, pathologists, and oncologists should be aware of the consequences associated with a delay in the diagnosis and/or initiation of therapy for Mullerian adenosarcoma with sarcomatous overgrowth. Although rare, this entity should be kept in the differential of young women presenting with abnormal menstrual bleeding in the presence of a pelvic mass.

## Competing interests

The author(s) declare that they have no competing interests.

## Authors' contributions

MF: data collection, literature review, paper writing.

EH: literature review, paper writing.

GM: pathology reading and revision, paper writing.

AN: literature review, paper editing.

All authors revised and approved the final draft of the manuscript.
